# Factors Contributing to Coronary Microvascular Dysfunction in Patients with Angina and Non-Obstructive Coronary Artery Disease

**DOI:** 10.3390/jcdd11070217

**Published:** 2024-07-10

**Authors:** Hiroki Teragawa, Yuko Uchimura, Chikage Oshita, Yu Hashimoto, Shuichi Nomura

**Affiliations:** Department of Cardiovascular Medicine, JR Hiroshima Hospital, 3-1-36, Futabanosato, Higashi-Ku, Hiroshima 732-0057, Japan; ymakita@hotmail.com (Y.U.); chikage-ooshita@jrhh.or.jp (C.O.); yu-hashimoto@jrhh.or.jp (Y.H.); shuuichi-nomura@jrhh.or.jp (S.N.)

**Keywords:** coronary microvascular dysfunction, coronary spasms, vasospastic angina

## Abstract

Background: Coronary microvascular dysfunction (CMD), characterised by a reduced coronary flow reserve (CFR) or an increased index of microcirculatory resistance (IMR), has received considerable attention as a cause of chest pain in recent years. However, the risks and causes of CMD remain unclear; therefore, effective treatment strategies have not yet been established. Heart failure or coronary artery disease (CAD) is a risk factor for CMD, with a higher prevalence among women. However, the other contributing factors remain unclear. In this study, we assessed the risk in patients with angina and non-obstructive coronary artery disease (ANOCA), excluding those with heart failure or organic stenosis of the coronary arteries. Furthermore, we analysed whether the risk of CMD differed according to component factors and sex. Methods: This study included 84 patients with ANOCA (36 men and 48 women; mean age, 63 years) who underwent coronary angiography and functional testing (CFT). The CFT included a spasm provocation test (SPT), followed by a coronary microvascular function test (CMVF). In the SPT, patients were mainly provoked by acetylcholine (ACh), and coronary spasm was defined as >90% transient coronary artery constriction on coronary angiography, accompanied by chest pain or ischaemic changes on electrocardiography. In 15 patients (18%) with negative ACh provocation, ergonovine maleate (EM) was administered as an additional provocative drug. In the CMVF, a pressure wire was inserted into the left anterior descending coronary artery using intravenous adenosine triphosphate, and the CFR and IMR were measured using previously described methods. A CFR < 2.0 or IMR ≥ 25 was indicative of CMD. The correlations between various laboratory indices and CMD and its components were investigated, and logistic regression analysis was performed, focusing on factors where *p* < 0.05. Results: Of the 84 patients, a CFR < 2.0 was found in 22 (26%) and an IMR ≥ 25 in 40 (48%) patients, with CMD identified in 46 (55%) patients. CMD was correlated with smoking (*p* = 0.020) and the use of EM (*p* = 0.020). The factors that correlated with a CFR < 2.0 included the echocardiograph index E/e′ (*p* = 0.013), which showed a weak but positive correlation with the CFR (r = 0.268, *p* = 0.013). Conversely, the factors correlated with an IMR ≥ 25 included RAS inhibitor usage (*p* = 0.018) and smoking (*p* = 0.042). Assessment of the risk of CMD according to sex revealed that smoking (*p* = 0.036) was the only factor associated with CMD in men, whereas the left ventricular mass index (*p* = 0.010) and low glycated haemoglobin levels (*p* = 0.012) were associated with CMD in women. Conclusions: Our results indicated that smoking status and EM use were associated with CMD. The risk of CMD differed between the two CMD components and sex. Although these factors should be considered when treating CMD, smoking cessation remains important. In addition, CMD assessment should be performed carefully when EM is used after ACh provocation. Further validation of our findings using prospective studies and large registries is warranted.

## 1. Introduction

Ischaemic heart disease without significant coronary artery stenosis includes angina with non-obstructive coronary artery disease (ANOCA), ischaemia with non-obstructive coronary artery disease, and myocardial infarction with non-obstructive coronary artery disease (MINOCA). These diseases are frequently encountered in clinical practice and have gained considerable attention in recent years [1,2,3,4].

Coronary microvascular dysfunction (CMD) is a known risk factor for ischaemic heart disease. CMD comprises two components: a low coronary flow reserve (CFR) and a high index of microcirculatory resistance (IMR). CMD can occur independently and in conjunction with coronary artery disease (CAD) with organic stenosis [5] or vasospastic angina (VSA) [6], and it is considered a poor prognostic factor. CMD has also been reported to be closely associated with all the subtypes of MINOCA [7]. Lifestyle management and beta-blockers are recognised treatments for CMD [2]; however, managing symptoms in patients with CMD remains challenging in clinical settings. CMD is more common in women and in patients with heart failure [8,9,10]; however, the association varies depending on the specific target population. Furthermore, the risk factors associated with CMD in women at higher risk remain unclear. Moreover, it is essential to understand whether the risks of CMD are comparable between patients with a low CFR and a high IMR.

Therefore, our study aimed to evaluate the risk factors stratified by sex and the components of CMD among patients with ANOCA diagnosed using the coronary function test (CFT), including the spasm provocation test (SPT) and coronary microvascular function test (CMVF), at our institution.

## 2. Materials and Methods

### 2.1. Study Population

**Figure 1:**
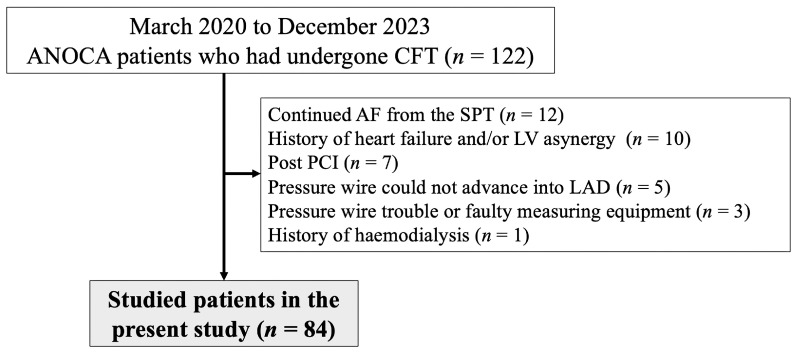
Study flow chart. AF, atrial fibrillation; ANOCA, angina with non-obstructive coronary artery disease; CFT, coronary function test; LAD, left anterior descending coronary artery; LV, left ventricular; PCI, percutaneous coronary intervention; SPT, spasm provocation test.

This retrospective study included 122 patients with chest pain who underwent coronary angiography (CAG) and CFT between March 2020 and December 2023. Patients with moderate coronary stenosis (% stenosis ≥ 50%), moderate chronic kidney disease (CKD) with an estimated glomerular filtration rate (eGFR) of <45 mL/min/1.73 m^2^, or with a history of heart failure or percutaneous coronary intervention (PCI) were excluded, as were patients with several risk factors (Figure 1). Finally, 84 patients who were diagnosed with ANOCA were included. The ethics committee of the JR Hiroshima Hospital approved this study (2023-52). Informed consent was obtained from all the included patients.

### 2.2. CFT

The SPT techniques used at our institution are based on previously published studies [11]. The SPT was performed following conventional diagnostic coronary angiography (CAG) using a percutaneous brachial or radial approach with a 5 Fr sheath and a diagnostic Judkins-type catheter. Following the initial CAG, the left coronary artery (LCA) was infused with 50, 100, and 200 µg of ACh for 20 s, with a 3 min interval between each infusion. Immediate CAG was performed either after the maximum infusion of ACh or after the induction of coronary spasms. Following the induction of spasms in the LCA, doses of 20, 50, and 80 µg of ACh were administered into the right coronary artery (RCA) for 20 s, with 3 min intervals between each dose. If coronary spasm occurred but spontaneously resolved during SPT for the RCA, intracoronary nitroglycerine (NTG) was not administered to the LCA. Conversely, if the infusion of ACh into the LCA caused long-lasting coronary spasms or resulted in unstable haemodynamics, 0.3 mg was administered directly to the coronary artery. Ergonovine maleate (EM) was administered intracoronarily to 15 patients (18%) who exhibited negative reactions to ACh provocation, as previously described [12,13]. The decision to administer EM was at the discretion of the attending physician. Following the completion of all the provocation tests for the LCA and RCA, CAG was repeated following the injection of NTG into the coronary artery. If the subsequent SPT yielded a negative result after NTG administration, the outcome was categorised as ‘unable to determine’ (NA).

The techniques used for the CMVF are described in detail in a previous study [14]. A PressureWire X Cabled Guidewire (Abbot Laboratories, Abbot Park, IL, USA) was used in conjunction with a pressure–temperature sensor tip. A RadiAnalyzerTM Xpress (Abbott Vascular, Santa Clara, CA, USA) was used to evaluate the parameters. The PressureWire was secured to the distal segment of the LAD, and three 3 mL injections of saline were administered at room temperature to generate a thermodilution curve to measure the resting mean transit time (Tmn). Adenosine triphosphate was intravenously infused into the peripheral veins at a rate of 160 μg/kg/min to induce hyperaemia. The proximal aortic pressure (Pa), distal arterial pressure (Pd), and Tmn were measured at maximum hyperaemia. The fractional flow reserve (FFR) was determined by calculating the lowest average of three successive beats under stable hyperaemia. The ratio between the resting Tmn and the hyperaemic Tmn was used to assess the CFR. The IMR was determined during hyperaemia using the formula Pd × Tmn. Before monitoring the readings in each coronary artery, we calibrated the aortic pressure in the catheter and the pressure obtained using the PressureWire to mitigate the pressure drift. We ensured that there was no discrepancy between the pressure measured after removing the PressureWire and the aortic pressure.

### 2.3. Definitions of CFT

The procedure for determining the coronary artery diameter has previously been described [15]. Segments with spasticity and atherosclerosis were selected for the quantitative analysis. The analysis was performed using the average values obtained from the three measurements. The percentage deviation from the initial angiographic data was used to express the changes in the coronary artery diameter in response to the ACh and NTG infusions. Atherosclerotic lesions were defined as those with more than 20% stenosis. The presence of myocardial bridging, defined as a systolic reduction of >20% in the coronary artery diameter, was investigated [11].

Coronary spasm was characterised by the constriction of the epicardial coronary arteries by more than 90%, as observed on angiography during the SPT. Moreover, the presence of identifiable chest pain and/or abnormal ST-segment deviation on electrocardiography (ECG) is indicative of coronary spasm [3,16]. According to the American Heart Association, focal spasm is defined as the transient constriction of a blood vessel by more than 90%, which only occurs within the confines of a single isolated coronary segment [17]. Diffuse spasm is a condition in which vasoconstriction of the coronary arteries occurs in two adjacent segments, affecting more than 90% of the arteries [17]. The time at which subsequent SPT results became negative after the necessary application of NTG to one coronary artery could not be ascertained. The study classified the dosages of ACh as low (L), moderate (M), and high (H), corresponding to 50, 100, and 200 µg for the LCA, and 20, 50, 80 µg for the RCA, respectively. Microvascular spasm (MVS) refers to the absence of angiographic coronary spasms accompanied by characteristic chest pain and ST-T ECG changes during SPT [18]. CMD was defined as an IMR of ≥25 units or a CFR < 2.0 [1].

### 2.4. Definitions of Clinical Parameters

Based on their smoking status, the patients were classified as active smokers, former smokers (who had stopped smoking for at least one month), or never smokers. A family history of coronary artery disease (CAD) was ascertained from the medical history of the patient and family members, electronic medical records, and referral letters. The pertinent medication history at the time of admission was also examined [19]. Fasting blood and urine tests were performed on the morning of the CAG. Hypertension, dyslipidaemia, diabetes mellitus, and metabolic syndrome (MtS) were identified based on previously described criteria [20]. The glomerular filtration ratio (GFR, mL/min/1.73 m^2^) was calculated using the standard formula, and CKD was diagnosed using the standard criteria [21]. On echocardiography (UCG), the left ventricular ejection fraction (LVEF) was measured using the modified Simpson’s method. The left ventricular mass index (LVMI) was calculated using the formula of Devereux and Reichek [22,23]. As an index of the left ventricular diastolic function, the peak early diastolic velocity (E)/early diastolic (e’) peak velocity at the septal side ratio was assessed [24].

### 2.5. Statistical Analyses

Continuous data were expressed as means with standard deviations. Non-normally distributed data were logged and analysed as continuous variables. Student’s unpaired *t*-test, the Wilcoxon signed-rank test or χ^2^ analysis was used to compare the baseline characteristics of the groups and the results of the CAG and CFT. Spearman’s rank correlation coefficient was used to analyse the correlation between the CFR, IMR, and clinical parameters. Logistic regression analysis was performed to determine the presence of CMD and clinical parameters, including factors with a *p*-value < 0.05 in the present study. The analysis was performed using factors with complete data. These analyses were performed for the entire study population stratified according to sex. All the statistical analyses were performed using JMP Ver. 17 (SAS Institute Inc., Cary, NC, USA). Statistical significance was set at *p* < 0.05.

## 3. Results

### 3.1. Patients’ Characteristics

This study included 36 men (42%) and 48 women (58%). Of the 84 patients, a CFR < 2.0 and an IMR ≥ 25 were observed in 22 (26%) and 40 (48%) patients, respectively, and CMD was detected in 46 patients (55%). Table 1 lists the clinical parameters of the patients with and without CMD. The proportion of smokers was higher in the CMD group than in the non-CMD group (*p* = 0.032), and the number of women (*p* = 0.099) and those taking renin–angiotensin system (RAS) inhibitors (*p* = 0.059) was higher than in the non-CMD group. The haemoglobin (*p* = 0.097) and low-density lipoprotein (LDL) cholesterol (*p* = 0.052) levels were lower in the CMD group than in the non-CMD group. Regarding the CFT results, the frequency of EM use was significantly higher in the CMD group than in the non-CMD group (*p* = 0.030). The frequency of VSA did not differ between the two groups (*p* = 0.117), and the values of Pd/Pa at baseline and FFR were similar between the two groups. In the CMD group, the CFR was significantly lower and the IMR was significantly higher than those in the non-CMD group (both *p* < 0.001).

To determine the factors contributing to CMD in all the included patients, logistic regression analyses were performed using factors with *p* < 0.05. Smoking status (*p* = 0.020) and EM use (*p* = 0.020) were significantly associated with the presence of CMD (Table 2A).

### 3.2. Clinical Parameters Associated with CFR and IMR

**Figure 2:**
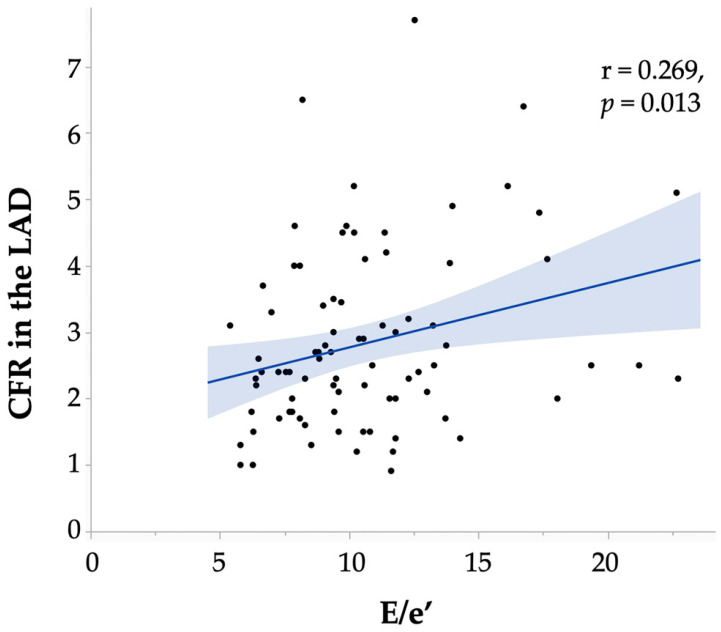
Relationship between the E/e′ and the CFR in the LAD. The straight line is the regression line and the confidence interval is shown in blue. CFR, coronary flow reserve; LAD, left anterior descending coronary artery.

The IMR was negatively associated with the CFR (r = −0.411, *p* < 0.001). The clinical parameters associated with the CFR included smoking (CFR: 2.1 ± 0.6 in 12 smokers, 3.0 ± 1.4 in 72 non-smokers, *p* = 0.048) and the value of E/e′ (r = 0.268, *p* = 0.013, Figure 2, Supplementary Table S1). Logistic regression analysis showed that the E/e′ value was the significant factor associated with a reduced CFR (<2.0, *p* = 0.020, Table 2B).

**Figure 3:**
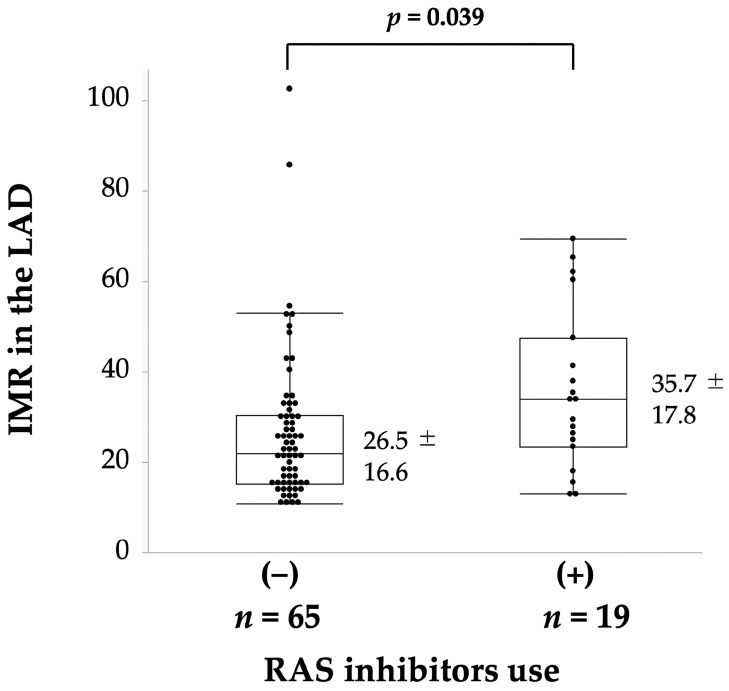
Values of the IMR in relation to RAS inhibitor use. IMR, index of microcirculatory resistance; LAD, left anterior descending coronary artery; RAS, renin–angiotensin system.

Regarding the factors influencing the IMR, body mass index (r = 0.284, *p* = 0.009), smoking history (IMR: 44.2 ± 22.3 in 12 smokers, 26.0 ± 14.9 in 72 non-smokers, *p* < 0.001), HDL cholesterol level (r = −0.219, *p* = 0.046) and RAS inhibitor use (IMR: 35.7 ± 17.8 in 19 patients on RAS inhibitors, 26.5 ± 16.6 in 65 patients not on RAS inhibitors, *p* = 0.039, Figure 3) were significant factors. Logistic regression analysis showed that RAS inhibitor use (*p* = 0.018) and smoking status (*p* = 0.041) were significantly associated with an increased IMR (≥25, Table 2C). Regarding the use of RAS inhibitors among the 19 patients included in this study, angiotensin-converting enzyme inhibitors and angiotensin II receptor blockers were used by 1 and 18 patients, respectively.

### 3.3. Clinical Parameters Associated with the Presence of CMD in Relation to Gender

Table 3 summarises the sex-specific clinical characteristics of the patients with and without CMD. Smoking was the only factor that contributed to CMD in men (*p* = 0.036). In women, univariate analyses showed that low HbA1c levels (*p* = 0.033), a low LVMI (*p* = 0.042), and EM use (*p* = 0.044) were associated with CMD. Logistic regression analysis revealed that the LVMI (*p* = 0.010) and low HbA1c levels (*p* = 0.012) were significantly associated with CMD in women (Table 2D).

## 4. Discussion

This study analysed the risk factors for CMD in patients with ANOCA who underwent SPT followed by CMVF. Of the 84 patients with ANOCA, 55% had CMD. Logistic regression analyses revealed that smoking status and EM use were associated with a higher risk of developing CMD. Various factors influenced the CMD components CFR < 2.0 and IMR ≥ 25—namely, the echocardiographic index E/e′ is associated with a low CFR, while the use of oral RAS inhibitors and smoking status are associated with a high IMR. The risk of CMD according to sex was related to smoking in men and low HbA1c levels and high LVMI levels in women. These findings suggest that the causes of CMD are diverse and that differences in CMD components and sex should be considered in the treatment of CMD.

It has been shown that pre-existing heart failure [8,9,10,25], coronary artery disease [9,26], or valvular disease such as aortic stenosis [27,28] contribute to CMD. To determine the clinical risk of CMD in patients with ANOCA, patients with heart failure or a history of coronary artery disease were excluded from the present study. In addition, patients with paroxysmal AF, which is a frequent complication of SPT [29] and may affect CMVF measurements, were excluded. The relationship between CMD and AF occurrence after the SPT should be investigated in future studies. Thus, we demonstrated that smoking status and EM use were significantly associated with CMD in the present study.

Our results showed that smoking was a risk factor for CMD in all the studied patients and was the only risk factor for men with ANOCA, which aligns with previous findings [20,30,31,32]. In our previous study [20], the IMR was comparable between past smokers and non-smokers. Smoking is a major risk factor for CMD and VSA [33], indicating that smoking cessation is essential.

EM has been shown to reduce CFR [34,35], especially in VSA [36], and the use of EM may be associated with the presence of CMD. It is possible that the occurrence of contractions at the arteriolar level led to an increased frequency of CMD. Our results supported this hypothesis. We consider the additional administration of EM to be a useful test for SPT [12,37]. It is unclear how long after EM provocation that coronary microcirculation improves or whether the sole use of EM for SPT may be associated with the presence of CMD; however, cardiologists should be aware that EM administration after ACh provocations may increase the CMD frequency.

This study analysed the factors individually associated with a CFR < 2.0 and an IMR ≥ 25, which are included in the definition of CMD. Our findings demonstrated that the CFR and IMR had moderately negative correlations; however, the factors associated with the aforementioned causes were different. The E/e′, an echocardiographic index, showed a weak but positive correlation with the CFR. The relationship between the E/e′ and CMD remains inconclusive, with studies demonstrating a correlation between a higher E/e′ and CMD in patients with heart failure with preserved ejection fraction [25,38,39], whereas others reported no correlation between the E/e′ and coronary microvascular function, as assessed by cardiac computed tomography in patients with ANOCA [40]. The causes and risks of CMD vary widely, and the results may differ depending on the patient population and the specific factors investigated. Moreover, the methods of measuring the CFR in the above studies [25,38,39,40], including non-invasive methods, vary across different studies. However, our findings elucidated a positive correlation between the CFR and the E/e′ among ANOCA patients, excluding those with obvious heart failure or cardiomyopathy. Although the cause or outcome of this association is unknown, one speculation is that, in patients with ANOCA and mildly reduced left ventricular diastolic function, a compensatory function that increases the CFR may exist. However, this study was based on a small number of patients, and the correlation coefficient was not high. Therefore, further validation is needed, either by increasing the number of patients or by using a large cohort.

We also showed that RAS inhibitor intake remained a significant risk factor in the presence of an IMR ≥ 25, which is one of the components of CMD. The IMR in the RAS inhibitor group was significantly higher than that in the non-RAS inhibitor group. Currently, many studies, including prospective studies, have investigated the effects of RAS inhibitors on the CFR, which improve the CFR [41,42,43,44] and have been listed in the Expert Consensus Document for treating CMD [2]. However, this was a cross-sectional study that examined the association between oral medication and CMVF upon hospital admission; the exact association, whether the cause or effect, could not be determined. Additionally, the type and pharmacological action of each RAS inhibitor differs, and it may not be possible to generalise their effects. However, the RAS inhibitor intake may have some influence on the increased IMR, and our results demonstrated no correlation between RAS inhibitor intake and the CFR. Despite the challenges associated with invasive testing, prospective studies may provide insights into the possible effects of RAS inhibitors on the IMR. In addition, the diagnosis of CMD is currently based on a CFR < 2.0 and/or an IMR ≥ 25. The frequency of each construct differs, with more people with an IMR ≥ 25, and these constructs may have different causes/risks, which should also be considered when assessing the cause/risk. The validity of a CMD diagnosis using these two factors, including reviewing the cut-off values for each factor, warrants further clarification in large-scale studies.

Several studies have reported that the risk of CMD is higher in women [8,45,46,47]. This could be attributed to anatomical differences in women, such as small coronary artery diameters and increased shear stress due to increased coronary artery blood flow [48], in addition to the presence of vascular endothelial dysfunction due to low oestrogen levels [49,50]. Our results did not show a high frequency of CMD among women. The present study included vasospastic angina (VSA) patients (69%, 58/84) and showed that VSA occurred more frequently in men than in women (Supplementary Table S2). Such specific characteristics of the study patients may have contributed to the different results. Nevertheless, the risk of CMD in women differs from that in men. Elevated LVMI and low HbA1c levels were associated with CMD in women. The LVMI is an indicator of left ventricular diastolic dysfunction and is implicated in CMD [51,52]. Although the frequency of beta-blocker use tended to be higher among women with CMD, the frequency of the intake of other antihypertensive medications, the comorbidity of hypertension, and body size did not differ between women with and without CMD. Postmenopausal oestrogen levels may also affect the left ventricular myocardium [53,54]. Although oestrogen levels were not measured, these factors may have influenced the combination of the LVMI and CMD in the women. In addition, low HbA1c levels contributed to CMD in women with ANOCA, despite no significant differences in the haemoglobin levels. Diabetes is a known risk factor for CMD [30]. However, the HbA1c level may not be a risk factor for CMD, as some studies have found no correlation between HgA1c and CFR in patients whether or not diabetic [55]. However, there is no clear explanation for the association between low HgA1c levels and CMD. Owing to the small sample size and the differences in the backgrounds of the patients included in this cross-sectional study, the influence of incidental results cannot be ruled out. Future prospective observational studies and multicentre studies are warranted to clarify the association between the HbA1c level and the CMD risk in women with ANOCA.

This study had several limitations. First, this was a retrospective single-centre study with a small sample size, which limits the generalisability of the findings. We considered the patient’s sex in the analysis, which resulted in an even smaller sample for the analysis and may have affected our results and conclusions. Second, because of the cross-sectional study design, the association between the clinical examination findings at admission and CMD or its associated factors could not be analysed. Although we examined the available data, we cannot rule out the possibility that such an association was obtained by chance because of the presence of factors that were not examined in this study. Future studies should clarify these relationships using prospective observational studies and multicentre participants to reduce the potential bias. Third, our study included several patients with VSA, all of whom were evaluated for CMVF after the SPT with ACh. ACh administration influences the CMVF values, especially in patients with VSA [56]. The results of the present study were obtained using CMVF from the SPT. Fourth, EM was administered to patients with negative ACh results at our institution. Although the guidelines do not recommend the additional use of EM [3], it is extremely useful in identifying coronary spasms due to the stimulation of the muscarinic receptors with acetylcholine and serotonin receptors, and no particular increase in complications has been reported [12,37]. The additional use of EM was approved by our ethics committee and was used when VSA was strongly suspected at our institution. However, the decision to administer EM was made at the discretion of the attending physician rather than according to a uniform protocol. Fifth, all the EM injections in this study were administered after ACh administration, and it was impossible to examine whether EM alone affected the CMD frequency. Finally, the vessel of interest in this study was the LAD; however, previous studies have indicated that the presence of CMD may differ between the LAD and other coronary arteries [20] and that CMD may be present in other vessels but not in the LAD. Therefore, our results may not apply to other vessels. However, CMVF can be performed in the LAD [4], and we believe that the results of this study evaluating CMD in the LAD may offer valuable information.

## 5. Conclusions

We investigated the frequency and risk of CMD in 84 patients with ANOCA, excluding those with heart failure or coronary artery disease. CMD was detected in 55% of the patients. Logistic regression analysis revealed smoking status and EM use were associated with the presence of CMD. The CMD components, a CFR < 2.0 and an IMR ≥ 25, were correlated with a lower E/e′, and RAS inhibitor intake and smoking status, respectively. When stratified by sex, smoking was identified as the major risk factor among men and high LVMI and low HgA1c levels among women. This finding underscores the importance of considering sex and abnormal CFR and IMR values when assessing the cause of CMD. Based on these results, smoking cessation should be considered as the first treatment attempt. In addition, much attention should be paid to the assessment of CMD when using EM in addition to ACh provocations. Further validation of our findings using prospective studies and larger cohorts is warranted.

## Figures and Tables

**Table 1:** Clinical parameters of patients with and without CMD.

Factors	CMD (−)	CMD (+)	*p* Value
No.	38 (45)	46 (55)	
Age (years)	63 ± 14	63 ± 14	0.852
Men/women	20/18	16/30	0.099
Body mass index	24.2 ± 4.6	24.6 ± 4.8	0.681
Coronary risk factors (%)			
Smoking history	2 (5)	10 (21)	0.032
Hypertension	22 (58)	31 (67)	0.369
Dyslipidaemia	16 (42)	23 (50)	0.470
Diabetes mellitus	6 (16)	6 (13)	0.720
Family history of CAD (%)	11 (29)	14 (30)	0.882
Presence of MtS (%)	9 (24)	6 (13)	0.205
Presence of CKD (%)	9 (24)	13 (28)	0.635
Biochemical parameters			
Haemoglobin (g/mL)	14.0 ± 1.2	13.5± 1.4	0.097
LDL cholesterol (mg/dL)	114 ± 29	101 ± 27	0.052
HDL cholesterol (mg/dL)	62 ± 17	62 ± 15	0.934
Triglycerides (mg/dL)	126 ± 77	112 ± 58	0.374
Fasting blood sugar (mg/dL)	105 ± 18	105 ± 18	0.866
HbA1c (%)	6.0 ± 0.6	5.9 ± 0.6	0.305
Log CRP	−1.28 ± 0.52	−1.28 ± 0.48	0.972
eGFR (mL/min/1.73 m^2^)	69.7 ± 14.3	69.7 ± 14.2	0.987
Log NT proBNP	1.78 ± 0.39	1.87 ± 0.40	0.269
Echocardiography			
LVEF (%)	66 ± 7	68 ± 5	0.298
LVMI (g/m^2^)	75 ± 19	82 ± 20	0.116
E/e′	10.3 ± 3.1	10.9 ± 4.2	0.437
Medication history (%)			
CCB	19 (50)	17 (37)	0.229
RAS inhibitors	5 (13)	14 (30)	0.059
Beta-blockers	2 (5)	6 (13)	0.227
Anti-platelet therapy	2 (5)	6 (13)	0.227
Lipid-lowering therapy	12 (32)	20 (43)	0.264
CAG CFT			
Atherosclerotic change (%)	14 (37)	12 (26)	0.289
Myocardial bridging (%)	10 (26)	10 (22)	0.624
Dose of ACh (L/M/H)	4/17/17	3/16/27	0.428
Use of EM (%)	3 (8)	12 (26)	0.030
VSA (%)	28 (74)	30 (65)	0.406
VSA type in the LAD	6/20/6/6	14/13/10/9	0.134
(Focal/diffuse/MVS/non-specific)			
Presence of focal spasm (%)	6 (15)	14 (30)	0.117
Baseline Pd/Pa	0.96 ± 0.02	0.96 ± 0.03	0.633
FFR	0.91 ± 0.04	0.92 ± 0.05	0.631
CFR	3.5 ± 1.2	2.2 ± 1.2	<0.001
CFR < 2.0	0 (0)	22 (48)	<0.001
IMR	16.6 ± 4.1	38.4 ± 17.7	<0.001
IMR ≥ 25	0 (0)	40 (87)	<0.001

Numbers are expressed as numbers (percentages) and values are expressed as the mean with standard deviation. ACh, acetylcholine; CAD, coronary artery disease; CAG, coronary angiography; CCB, calcium channel blocker; CKD, chronic kidney disease; CFR, coronary flow reserve; CMD, coronary microvascular dysfunction; CRP, C-reactive protein; CFT, coronary function test; eGFR, estimated glomerular filtration ratio; E/e′, echocardiograph index; EM, ergonovine maleate, FFR, fractional flow reserve; HDL, high-density lipoprotein; HbA1c, glycated haemoglobin; IMR, index of microcirculatory resistance; LAD, left anterior descending coronary artery; LDL, low-density lipoprotein; L/M/H, low/moderate/high dose of ACh; LVEF, left ventricular ejection fraction; LVMI, left ventricular mass index; MVS, microvascular spasm; MtS, metabolic syndrome; No., number; NT proBNP, N-terminal pro brain natriuretic peptide; Pa, aortic pressure; Pd, distal pressure; RAS, renin–angiotensin system; VSA, vasospastic angina.

**Table 2:** Results of the logistic regression analyses.

(A) Logistic Regression Analyses to Determine the Risk Factors for CMD in all the Patients			
Factors	Odds Ratio	CI	*p* Value
Smoking	5.45	1.28–37.66	0.020
Use of EM	4.45	1.25–21.15	0.020
R^2^ = 0.09			
**(B) Logistic Regression Analyses to Determine the Factors Associated with a CFR < 2.0 in all the Patients**			
**Factors**	**Odds Ratio**	**CI**	***p*** **Value**
E/e′	0.82	0.67–0.97	0.020
Smoking	2.05	0.53–7.54	0.288
R^2^ = 0.07			
**(C) Logistic Regression Analyses to Determine the Factors Associated with an IMR ≥ 25 in all the Patients**			
**Factors**	**Odds Ratio**	**CI**	***p*** **Value**
RAS inhibitors use	3.92	1.26–13.86	0.018
Smoking	4.15	1.05–21.06	0.042
Body mass index	1.04	0.94–1.17	0.447
Low HDL cholesterol levels	1.01	0.98–1.04	0.656
R^2^ = 0.102			
**(D) Logistic Regression Analyses to Determine the Risk Factors for CMD in Women**			
**Factors**	**Odds Ratio**	**CI**	***p*** **Value**
LVMI	1.05	1.01–1.09	0.010
Low HbA1c levels	0.13	0.02–0.66	0.012
Use of EM	5.25	0.76–105.8	0.097
R^2^ = 0.237			

CFR, coronary flow reserve; CI, confidence interval; CMD, coronary microvascular dysfunction; EM, ergonovine maleate; E/e′, echocardiograph index; HDL, high-density lipoprotein; HbA1c, glycated haemoglobin; IMR, index of microcirculatory resistance; LVMI, left ventricular mass index; RAS, renin–angiotensin system.

**Table 3:** Sex-specific differences in clinical parameters associated with CMD.

Factors	Men			Women		
	CMD (−)	CMD (+)	*p* Value	CMD (−)	CMD (+)	*p* Value
No.	20 (56)	16 (44)		18 (37)	30 (63)	
Age (years)	64 ± 13	62 ± 15	0.678	63 ± 15	64 ± 14	0.968
Body mass index	25.6 ± 4.8	25.9 ± 4.7	0.860	22.5 ± 3.9	23.9 ± 4.9	0.320
Coronary risk factors (%)						
Smoking history	1 (5)	5 (31)	0.036	1 (6)	5 (17)	0.260
Hypertension	11 (55)	11 (69)	0.400	11 (61)	20 (67)	0.697
Dyslipidaemia	7 (35)	10 (63)	0.101	9 (50)	13 (43)	0.654
Diabetes mellitus	3 (15)	3 (19)	0.764	3 (17)	3 (10)	0.499
Family history of CAD (%)	11 (29)	14 (30)	0.882	4 (22)	11 (37)	0.296
Presence of MtS (%)	8 (40)	6 (13)	0.205	1 (6)	3 (10)	0.590
Presence of CKD (%)	9 (24)	3 (19)	0.169	5 (28)	7 (23)	0.731
Biochemical parameters						
Haemoglobin (g/mL)	14.5 ± 1.3	14.3 ± 1.7	0.745	13.5 ± 0.9	13.1 ± 1.1	0.187
LDL cholesterol (mg/dL)	108 ± 26	95 ± 27	0.141	120 ± 32	105 ± 27	0.097
HDL cholesterol (mg/dL)	53 ± 11	53 ± 12	0.858	71 ± 18	66 ± 14	0.308
Triglyceride (mg/dL)	150 ± 94	128 ± 74	0.435	98 ± 39	112 ± 58	0.628
Fasting blood sugar (mg/dL)	106 ± 17	113 ± 21	0.266	103 ± 19	101 ± 14	0.697
HbA1c (%)	6.0 ± 0.8	6.0 ± 0.8	0.766	6.1 ± 0.5	5.8 ± 0.4	0.033
Log CRP	−1.18 ± 0.53	−1.28 ± 0.53	0.608	−1.38 ± 0.50	−1.28 ± 0.46	0.500
eGFR (mL/min/1.73 m^2^)	73.4 ± 13.8	68.9 ± 13.9	0.339	65.5 ± 14.1	70.1 ± 14.6	0.285
Log NT proBNP	1.78 ± 0.43	1.68 ± 0.39	0.458	1.77 ± 0.35	1.98 ± 0.37	0.062
Echocardiography						
LVEF (%)	65 ± 8	68 ± 6	0.158	68 ± 5	67 ± 6	0.730
LVMI (g/m^2^)	82 ± 19	84 ± 13	0.751	68 ± 17	81 ± 23	0.042
E/e′	10.1 ± 2.9	8.9 ± 2.3	0.155	10.4 ± 3.4	12.0 ± 4.7	0.215
Medication history (%)						
CCB	9 (45)	6 (38)	0.650	10 (56)	11 (37)	0.202
RAS inhibitors	3 (15)	6 (38)	0.121	2 (11)	8 (27)	0.199
Beta-blockers	2 (10)	1 (6)	0.686	0 (0)	6 (13)	0.067
Anti-platelet therapy	2 (10)	4 (25)	0.230	0 (0)	2 (7)	0.263
Lipid-lowering therapy	7 (35)	9 (56)	0.202	5 (28)	11 (37)	0.527
CAG CFT						
Atherosclerotic change (%)	9 (45)	7 (44)	0.940	5 (28)	5 (17)	0.364
Myocardial bridging (%)	5 (25)	4 (25)	1.000	5 (28)	6 (20)	0.535
Dose of ACh (L/M/H)	3/11/6	2/9/5	0.977	1/6/11	1/7/22	0.673
Use of EM (%)	2 (10)	3 (19)	0.451	1 (6)	9 (30)	0.044
VSA (%)	18 (90)	14 (88)	0.813	10 (56)	16 (53)	0.881
VSA type in the LAD	5/11/1/3	7/6/0/3	0.496	1/9/5/3	7/7/10/6	0.194
(focal/diffuse/MVS/non-specific)						
Presence of focal spasm (%)	5 (25)	7 (44)	0.236	1 (6)	7 (23)	0.110
Baseline Pd/Pa	0.95 ± 0.02	0.95 ± 0.03	0.820	0.96 ± 0.02	0.96 ± 0.03	0.732
FFR	0.90 ± 0.04	0.88 ± 0.04	0.302	0.92 ± 0.03	0.93 ± 0.05	0.520
CFR	3.7 ± 1.3	2.0 ± 0.7	<0.001	3.4 ± 1.1	2.4 ± 1.4	0.012
CFR < 2.0	0 (0)	7 (44)	0.001	0 (0)	15 (50)	<0.001
IMR	17.9 ± 4.3	41.3 ± 15.0	<0.001	15.1 ± 3.4	36.9 ± 19.0	<0.001
IMR ≥ 25	0 (0)	14 (88)	<0.001	0 (0)	26 (87)	<0.001

Numbers are expressed as numbers (percentages) and values are expressed as the mean with standard deviation. ACh, acetylcholine; CAD, coronary artery disease; CAG, coronary angiography; CCB, calcium channel blocker; CKD, chronic kidney disease; CFR, coronary flow reserve; CMD: coronary microvascular dysfunction; CRP, C-reactive protein; CFT, coronary function test; E/e′, echocardiograph index; eGFR, estimated glomerular filtration ratio; EM, ergonovine maleate; FFR, fractional flow reserve; HDL, high-density lipoprotein; HbA1c, glycated haemoglobin; IMR, index of microcirculatory resistance; LAD, left anterior descending coronary artery; LDL, low-density lipoprotein; L/M/H, low/moderate/high dose of acetylcholine; LVEF, left ventricular ejection fraction; LVMI, left ventricular mass index; MVS, microvascular spasm; MtS, metabolic syndrome; No., number; NT proBNP, N-terminal pro brain natriuretic peptide; Pa, aortic pressure; Pd, distal pressure; RAS, renin-angiotensin system; VSA, vasospastic angina.

## Data Availability

The original contributions presented in the study are included in the article/Supplementary Materials, further inquiries can be directed to the corresponding author.

## Supplementary Materials

The following supporting information can be downloaded at: https://www.mdpi.com/article/10.3390/jcdd11070217/s1, Table S1: Relationship between clinical parameters and CFR, IMR, and the presence of CMD; Table S2: Baseline characteristics of the included patients and sex-specific characteristics.
