# Supplementary material for: Factors Contributing to Coronary Microvascular Dysfunction in Patients with Angina and Non-Obstructive Coronary Artery Disease
Source: J Cardiovasc Dev Dis. 2024 Jul 10;11(7):217. doi: 10.3390/jcdd11070217 (PMC11277519; doi:10.3390/jcdd11070217)
Supplement: Supplementary file 1 [file jcdd-11-00217-s001.zip › jcdd-3040320-supplementary.pdf]

Supplementary file

Table S1. Relationship between clinical parameters and CFR, IMR, and the presence of CMD

Factors	Numbers (%) or values	Relationship between factors and CFR, *p* value	Relationship between factors and IMR, *p* value	Relationship between factors and CMD, *p* value
Age (years)	63 ± 14	0.339	0.551	0.850
Men/Women	36 / 48	0.469	0.906	0.099
Body mass index	24.4 ± 4.7	0.147	0.009	0.676
Coronary risk factors (%)				
Smoking history	12 (14)	0.041	< 0.001	0.032
Hypertension	53 (63)	0.480	0.261	0.369
Dyslipidemia	39 (46)	0.582	0.381	0.470
Diabetes mellitus	12 (14)	0.740	0.395	0.720
Family history of CAD (%)	25 (30)	0.214	0.272	0.882
Presence of MtS (%)	15 (18)	0.213	0.068	0.205
Presence of CKD (%)	22 (26)	0.439	0.586	0.773
Biochemical parameters				
Haemoglobin (g/mL)	13.8 ± 1.3	0.333	0.446	0.093
LDL-cholesterol (mg/dL)	107 ± 28	0.250	0.147	0.049
HDL-cholesterol (mg/dL)	62 ± 16	0.569	0.046	0.936
Triglyceride (mg/dL)	118 ± 67	0.236	0.498	0.367
Fasting blood sugar (mg/dL)	105 ± 17	0.893	0.595	0.864
HbA1c (%)	6.0 ± 0.6	0.280	0.780	0.299
Log CRP	-1.28 ± 0.50	0.433	0.230	0.972
eGFR (mL/min/1.73 m^2)	69.7 ± 14.2	0.383	0.485	0.987
Log NT-proBNP	1.83 ± 0.39	0.755	0.132	0.261
Echocardiography				
LVEF (%)	67 ± 6	0.890	0.277	0.291
LVMI (g/m^2)	79 ± 20	0.620	0.119	0.110
E/e'	10.6 ± 3.8	0.013	0.498	0.428
Medication history (%)				
CCB	36 (43)	0.298	0.683	0.229
RAS inhibitors	19 (23)	0.611	0.039	0.059
Beta-blockers	8 (10)	0.053	0.547	0.227
Anti-platelet therapy	8 (10)	0.308	0.150	0.227
Lipid-lowering therapy	32 (38)	0.397	0.227	0.264
CAG・CFT				
Atherosclerotic change (%)	26 (31)	0.158	0.883	0.203
Myocardial bridging (%)	20 (24)	0.478	0.897	0.289
Dose of ACh (L/M/H)	7/34/44	0.839	0.467	0.428
Use of EM (%)	15 (18)	0.804	0.154	0.030
VSA (%)	58 (69)	0.720	0.383	0.404

VSA type in the LAD (Focal/diffuse/MVS/non-specific)	20/33/16/15	0.558	0.075	0.134
Presence of focal spasm (%)	20 (24)	0.306	0.403	0.117
Baseline Pd/Pa	0.96 ± 0.02	0.655	0.455	0.628
FFR	0.91 ± 0.05	0.795	0.548	0.626
CFR	2.8 ± 1.4		<0.001	
CFR < 2.0	22 (26)			
IMR	28.6 ± 17.2	< 0.001		
IMR ≥ 25	40 (48)			
CMD (%)	46 (55)			

Numbers are expressed as numbers (percentages) and values are expressed as the mean with standard deviation. ACh, acetylcholine; CAD, coronary artery disease; CAG, coronary angiography; CCB, calcium channel blocker; CKD, chronic kidney disease; CFR, coronary flow reserve; CMD, coronary microvascular dysfunction; CRP, C-reactive protein; CFT, coronary function test; eGFR, estimated glomerular filtration ratio; EM, ergonovine maleate; FFR, fractional flow reserve; HDL, high-density lipoprotein; HbA1c, glycated haemoglobin; IMR, index of microcirculatory resistance; LAD, left anterior descending coronary artery; LDL, low-density lipoprotein; L/M/H, low/moderate/high doses of acetylcholine; LVEF, left ventricular ejection fraction; LVMI, left ventricular mass index; MVS, microvascular spasm; MtS, metabolic syndrome; No., number; NT proBNP, N-terminal pro brain natriuretic peptide; Pa, aortic pressure; Pd, distal pressure; RAS, renin-angiotensin system; VSA, vasospastic angina.

Table S2. Baseline characteristics of the included patients and sex-specific characteristics

**Factors**	**All patients**	**Men**	**Women**	*p* value
**Numbers (%)**	84	36 (43)	48 (57)	
Age (years)	63 ± 14	63 ± 14	64 ± 14	0.782
Men/Women (%)	36 / 48			
Body mass index	24.4 ± 4.7	25.8 ± 4.7	23.4 ± 4.5	0.021
Coronary risk factors (%)				
Smoking history	12 (14)	6 (17)	6 (13)	0.589
Hypertension	53 (63)	22 (61)	31 (65)	0.744
Dyslipidaemia	39 (46)	17 (47)	22 (46)	0.900
Diabetes mellitus	12 (14)	6 (17)	6 (13)	0.589
Family history of CAD (%)	25 (30)	10 (28)	15 (31)	0.731
Presence of MtS	15 (18)	11 (31)	4 (8)	0.009
Presence of CKD	22 (26)	10 (28)	12 (25)	0.775
Medication history (%)				

CCB	36 (43)	15 (42)	21 (44)	0.849
RAS inhibitors	19 (23)	9 (25)	10 (21)	0.652
Beta-blockers	8 (10)	3 (8)	5 (10)	0.748
Anti-platelet therapy	8 (10)	6 (17)	2 (4)	0.053
Lipid-lowering therapy	32 (38)	16 (44)	16 (33)	0.300
Haemoglobin (g/dL)	13.8 ± 1.3	14.4 ± 1.4	13.3 ± 1.0	< 0.001
Presence of VSA (%)	58 (69)	32 (89)	26 (54)	< 0.001

Numbers are expressed as numbers (percentages) and values are expressed as the mean with standard deviation. CAD, coronary artery disease; CCB, calcium-channel blockers; CKD, chronic kidney disease; MtS, metabolic syndrome; RAS, renin-angiotensin system; VSA, vasospastic angina.
